# Sex differences in neuro(auto)immunity and chronic sciatic nerve pain

**DOI:** 10.1186/s13293-020-00339-y

**Published:** 2020-11-12

**Authors:** Katja Linher-Melville, Anita Shah, Gurmit Singh

**Affiliations:** 1 Department of Pathology and Molecular Medicine, McMaster University, Hamilton, Ontario Canada; 2 Michael G. DeGroote Institute for Pain Research and Care, McMaster University, Hamilton, Ontario Canada

**Keywords:** Chronic pain, X chromosome inactivation, Sex difference, Autoimmune, Immune system, Nociceptor, Gonadal hormone

## Abstract

Chronic pain occurs with greater frequency in women, with a parallel sexually dimorphic trend reported in sufferers of many autoimmune diseases. There is a need to continue examining neuro-immune-endocrine crosstalk in the context of sexual dimorphisms in chronic pain. Several phenomena in particular need to be further explored. In patients, autoantibodies to neural antigens have been associated with sensory pathway hyper-excitability, and the role of self-antigens released by damaged nerves remains to be defined. In addition, specific immune cells release pro-nociceptive cytokines that directly influence neural firing, while T lymphocytes activated by specific antigens secrete factors that either support nerve repair or exacerbate the damage. Modulating specific immune cell populations could therefore be a means to promote nerve recovery, with sex-specific outcomes. Understanding biological sex differences that maintain, or fail to maintain, neuroimmune homeostasis may inform the selection of sex-specific treatment regimens, improving chronic pain management by rebalancing neuroimmune feedback. Given the significance of interactions between nerves and immune cells in the generation and maintenance of neuropathic pain, this review focuses on sex differences and possible links with persistent autoimmune activity using sciatica as an example.

## Background

To improve the quality of life for many patients who cope daily with pain, appropriate disease-modifying strategies that examine biological risk factors, such as age, genetics, and sex, need to be universally implemented. This is especially pertinent, given that the physiological systems that are perturbed in a state of persistent pain involve complex networks, including the nervous and immune systems, endocrine feedback, and the vasculature. In order to significantly improve health outcomes, not only for pain but also for a range of conditions, a growing consensus is that biological sex differences must be addressed, across scientific disciplines [1]. A holistic view will provide an improved understanding of how pain develops and becomes chronic, and how men and women respond differently to nociceptive signaling as well as to treatments aimed at curbing signals that become aberrant.

A large-scale longitudinal study of over 45,000 participants in 16 European countries examined the prevalence of chronic pain over a lifespan. While its incidence increased in both sexes during aging, the numbers of men and women reporting chronic pain progressively diverged, with this separation commencing in early adulthood (http://www.painineurope.com; [2]). A plethora of studies support that there are higher rates of pain [3–7] and post-traumatic stress [8, 9] in women compared with men. Although spanning beyond the emphasis of the current review, literature supports that chronic pain represents a state of stress, and may be a significant factor in determining the incidence of depression, with coexistence of pain and depression in turn exacerbating the severity of each condition [10]. Interestingly, depression also occurs more frequently in women [11], and there may be shared biological mechanisms impacted by gonadal steroid hormones and the chromosomal complement that underlie these sex differences. Females also consistently report a greater sensitivity to muscle, pressure, and temperature-associated pain than males [6]. In relevant animal models, increased hypersensitivity [12, 13] and insufficient fear extinction [14, 15] have been reported in females, with experimental data supporting that thresholds and sensitivity are affected by the stage of the murine estrous cycle [16, 17].

These outcomes in females may be associated with over-active immune cell infiltration into damage-associated sites, with immune activity not returning to a homeostatic state post-injury. Of note, general sex differences in the human immune system of a healthy middle-aged population reflect a significantly higher number of peripheral T lymphocytes in women compared with men [18], with evidence supporting heightened adaptive immune responses in women [19, 20]. This “basal” sexually dimorphic immune system priming, which occurs in the absence of injury or insult, may influence the outcome of pathological conditions, including autoimmune disorders and persistent pain. It should be noted that there are significant connections between unresolved pain and neurological disorders other than stress sensitivity and depression, such as Alzheimer’s disease, Parkinson’s disease, and autism spectrum disorders [21–23]. While a discussion of these conditions is beyond the scope of this review, it is important to acknowledge that these conditions may provide important parallel, biologically relevant insights into neuroimmune sex differences that may also be applicable to pain research and the development of tailored analgesic therapies.

As clinical practices move toward addressing the distinct analgesic needs of males and females, it will be important to consistently account for the interconnected effects of both gonadal hormones and their associated pathways as well as the sex chromosomal status of individuals. It is now well-recognized that the activity of the peripheral and central nervous systems (PNS and CNS, respectively) are affected by the immune system, the state of which may dictate the efficacy of treatment outcomes. While perception and environment play important roles in nociception, this review discusses examples of specific, evolutionarily preserved, biological parameters that may differentially contribute to the persistence of pain in males and females. An emphasis is placed on impingement of the sciatic nerve, which represents a common injury that, in many cases, leads to the development of chronic neuropathic pain. Neuropathic pain, a complex condition that is often refractory to currently available treatment options, may arise from injury to the somatosensory system. Such an injury is not always sufficient to generate persistent pain, with age, genetic background, and sex all having been shown to shape an individual’s risk [24]. An emerging subcategory of pain research is focusing on the possibility that persistent neuropathic pain may have an underlying autoimmune component, especially given that, in general, both autoimmune and chronic pain disorders occur at higher rates in females [25–28].

Numerous hypotheses have been proposed to explain the mechanisms that underlie sex differences in pain signaling. In females, the immune system may be continuously “ready for action,” potentially due to the presence of two X chromosomes, each bearing a plethora of genes involved in immune responsiveness. Another potential explanation as to why women exhibit heightened immune responses that may drive chronic pain is the pregnancy-compensation hypothesis. This line of thinking proposes that during a woman’s reproductive years, the immune system is constantly readying itself for the “foreign” placenta. Declining parity rates could have repercussions in the event of nerve injury, such as an increased incidence in persistent pain, or less pronounced improvements in response to existing analgesics. In addition, gonadal hormones, including estradiol and testosterone, have also been implicated. Estradiol is both neuroprotective and neurodegenerative, with reproductive cycle and age-dependent outcomes. Testosterone elicits immunosuppressive effects, including maintenance of basal lymphocyte profiles that influence inflammatory responses to aberrant signals, such as neuronal damage-associated antigen presentation and immune cell activation. Several autoimmune diseases have been associated with lower-than-normal androgen levels, although the decline in testosterone during male aging and the effect of hormone replacement on chronic pain remain understudied. Each of these facets will be examined in the context of a peripheral nerve injury based on the current literature.

## Persistent sciatic nerve pain

Sciatica, a common type of neuropathic pain attributed to impingement of, or injury to, one or both of the sciatic nerves, is experienced by up to 10% of patients with chronic lower back pain, with a reported lifetime prevalence that ranges from 49 to 70% [29]. Importantly, lower back pain is among the types of pain reported to occur more frequently in women than in men [6]. The sciatic nerves are of mixed-function, consisting of both motor and sensory axons. They branch to peripherally innervate the legs, several muscles, and skin in the lower extremities [30], and their nociceptive component, represented by the sensory axons that include Aβ, Aδ, and C fibers, is associated with the dorsal root ganglia (DRG). A discussion of motor neurons, their ventral efferent axons (the ventral root), and their relationship to sciatic nerve pain is beyond the scope of the current review, which has instead focused on cross-talk between afferent nociceptors and immune cells. DRG neurons, which are peripheral nerve bundles that also contain the somata of nociceptors, convey pain signals from the periphery into the CNS (reviewed in [31]). Studies also suggest that DRG actively participate in nerve injury associated with platelet-activating factor (PAF), inflammation, and the development of neuropathic pain [32–35] by metabolically influencing functionally relevant pathways between the PNS and CNS. A deeper understanding of the role of DRG in the context of neuroimmune cross-talk may advance treatment options for persistent neuropathic pain. In support of this notion, according to a 2017 study by Deer et al., DRG stimulation was shown to relieve pain more effectively than stimulating the spinal cord in patients with complex regional pain syndrome [36], which presents with many of the same symptoms as sciatica and is discussed later in the current review. As the most common symptom of sciatica, peripheral neuropathic pain typically extends through the hip and buttock down one leg, with the leg consequently feeling numb, weak, or “tingly.” Preclinical studies based on sciatic nerve injury in rodents are frequently used to cost-effectively provide a model of neuropathic pain [37] and are therefore being used to examine sexually dimorphic outcomes. Indeed, behavioral and electrophysiological research by our group has shown that this type of pain persists in female rodents, while males respond well to various agents that have been tested to date [38–40].

### Neuronal signaling, reviewed

The primary focus for gaining a better mechanistic understanding of how sex influences pain has been at the level of neuronal signaling. Nociceptive nerve terminals express a variety of channels, molecules, and receptors, including ion channels, neuropeptides, and cytokine receptors, respectively (reviewed in [41]). Under normal circumstances, nociceptors detect potentially harmful stimuli, such as changes in pressure, noxious chemicals, and temperature. In response to a nerve injury that is signaled to be a homeostatic threat, nociceptors may become aberrantly activated, or hyper-excited, even in the continued absence of the initial stimulus that evoked pain. This provides the potential for a normally non-noxious stimulus, such as gentle stroking of the skin, to cause exquisite pain.

The coordination of a regenerative response requires that information about the peripheral nerve injury be relayed to the relevant soma (cell body) of the nociceptor, which, in the case of the sciatic nerve, occurs at the DRG (reviewed in [42]). Ascending pain signaling involves the transmission of nerve impulses, in the form of action potentials produced by excitable peripheral nociceptive neurons, along their axons to the DRG, with relay of pro-nociceptive messages towards second-order neurons located in the dorsal horn of the spinal cord, and further transmission to the thalamus, cortex, and ultimately, higher centers in the brain for further processing and initiation of relevant descending signals (reviewed in [42]). Physiologically, as succinctly described by Hammond, “an action potential is generated when the membrane potential of a specific cell location rapidly rises and falls: this depolarization then causes adjacent locations to similarly depolarize. The action potential is therefore merely a sudden and transient depolarization of the membrane.” And, “in neuronal somas and axons, action potentials have a large amplitude and a small duration: these are the Na^+^-dependent action potentials” [43]. As a “signal,” an action potential therefore represents the coordinated movement of sodium (Na^+^) and potassium (K^+^) ions across the membrane of a nerve cell, thereby altering its resting potential. The balance of these particular ions is shifted in response to a disturbance or injury, which may be chemical, electrical, or mechanical in nature, triggering changes in the net charge of the membrane. As mentioned above, it is important to note that the sequence of depolarization and repolarization events occurs in a localized area of the membrane. In the case of injury to the sciatic nerve, an aberrant, “non-physiological” action potential may be generated anywhere along the axon that is affected by the insult, which is then compounded further by the resulting immune response. These changes are then passed on to the next area of the membrane, along the entire length of the axon. In this manner, the action potential, as the nerve impulse or “signal,” is transmitted to the DRG.

It should be pointed out here that the conduction of an action potential may be modulated by the T-junction, the bifurcation point at which the peripheral axon of a sensory neuron separates into the central branch to continue on to the spinal cord, and the stem, which joins the cell body of the nerve within the DRG [44]. This pseudounipolar structural arrangement suggests a possibility for low-pass filtering in modeling studies [45], with impedance mismatch altering spike propagation (reviewed in [46]). This in turn would lead to variations in membrane potential and action potential conductance in proximity to the T-junction, potentially affecting the sensory information that is passed on into the CNS. Experimental evidence from unmyelinated and myelinated sensory neurons supports that spikes do fail as they pass through the DRG, most likely at the T-junction [47–49]. Studies have been conducted to model how the signaling of C-fibers, which represent a major type of nociceptor, is influenced by T-junction morphology in conjunction with local ion channel expression, as both may be important in pain signaling [50].

Hyper-excitability of nociceptors may arise due to continued aberrant action potential generation (changes in amplitude and/or frequency), disinhibition of synaptic transmission, a loss of synaptic connectivity, and the formation of new synaptic circuits [24], eventually manifesting itself as persistent pain. The process of central sensitization, which has been described as “an enhancement in the function of neurons and circuits in nociceptive pathways caused by increases in membrane excitability and synaptic efficacy as well as to reduced inhibition” [51], is a driver in the maintenance of chronic pain. Hyper-excitability may be influenced by distinct hormonal profiles in males and females. For instance, N-methyl-d-aspartate receptor (NMDAR) activation, which has been associated with a hyper-excited state [52], may occur in a sexually dimorphic manner. NMDAR currents in the DRG are more dense in female than in male rats, likely through a mechanism involving 17-β-estradiol [53]. In addition, certain DRG gene products responsible for ion transport, some of which may be involved in generating a hyper-excitable response, have been shown to be upregulated in female rats [54].

### Neuronal signal transduction mechanisms

It has long been recognized that numerous mediators released by diverse peripheral cell types (fibroblasts, immune cells, and neurons) such as bradykinin, cytokines (i.e., interleukin (IL)6; IL-6), free radicals, histamine, neurotrophins (i.e., NGF), peptides (i.e., substance P), prostanoids (i.e., PGE2), and protons can act directly on sensory nerve terminals [55]. In addition to nociceptor sensitization, these factors are able to stimulate the release of other substances, activate the immune system, and play a role in vasodilatation and plasma extravasation. Sensitization of the nociceptive system involves the binding of these mediators to receptors present on sensory neurons, resulting in activation of second-messenger pathways and modulation of ion channels. Some of these mediators also alter gene expression profiles of the nociceptor [56]. To reiterate, not only are responses accompanied by transient modifications related to the excitation and sensitization of afferent peripheral sensory terminals (reviewed in [57, 58]), they may also elicit more enduring changes in phenotype, which may be important for conditions in which persistent pain occurs. With regard to the latter, for example, phenotypic switching of specific nociceptive fiber types has been documented [59, 60], and phenotypic changes are elicited by nerve growth factor (NGF) during tissue inflammation; these changes include an increase in neuropeptide levels that in turn may amplify sensory input signals at the level of the spinal cord, increased peripheral neuroinflammation, and upregulation of growth-related substances that promote axonal sprouting in the area of the injury, culminating in a decrease in the overall excitability threshold [61]. Coupled with increased excitability of spinal neurons [62], specific neuronal genes, particularly microRNAs such as the miR-183 cluster [60], may be associated with allodynia and hyperalgesia.

Various mechanisms underlie signal transduction from the axon to the DRG soma (reviewed in [63]). As already mentioned, a large depolarizing voltage is relayed to the somata in response to a peripheral insult, resulting in spiking activity and sustained membrane depolarization involving neuronal voltage-gated Na^+^ channels, which ultimately leads to significant calcium influx in the axon and soma (reviewed in [63]). Changes in intracellular calcium levels are known to play an important role not only in neuronal signaling, but in gene expression [64], with the latter associated with a modification of the nociceptor phenotype.

Another process, referred to as positive injury signaling, is facilitated by the transport of kinases—in particular, members of the mitogen-activated protein kinase (MAPK) family such as c-Jun N-terminal kinase (JNK) and extracellular signal–regulated kinase (ERK)—which likely interact with dynein/dynactin in order to be transported to the DRG (reviewed in [63]). In this manner, these kinases are then able to regulate gene expression at the nuclear level within the cell bodies of the DRG.

There is evidence to suggest that signaling endosomes facilitate the transport of nerve growth factor (NGF) signals from nociceptive neurons to their somata [65]. This latter type of “nerve signaling” represents an interesting means by which a distal factor known to be associated with inflammation [66], produced and released by peripheral tissue (for example, fibroblasts rapidly produce upregulated levels of NGF in response to pain-inducing cutaneous plantar [67] and deep muscle [68] incision in rats, and it is also expressed by central and peripheral nerves, as well as microglia and peripheral immune cells [69]), is able to regulate the structure and function of sensory neurons. It should also be pointed out that, depending on where it is released, NGF diffuses to either peripheral sensory nerve endings or presynaptic axon terminals in the dorsal horn of the spinal cord, binding to and activating its cognate receptors [69]. Relevantly, Eskander et al. showed that NGF treatment induced a long-lasting increase in peripheral and central transient receptor potential (TRP) vanilloid 1 (TRPV1) activity in rodents, supported by increased capsaicin-mediated nociceptive responses, increased calcitonin gene-related peptide (CGRP) release from biopsies of the cutaneous hindpaw, as well as increased capsaicin-evoked inward current and upregulated membrane expression of TRPV1 protein in DRG neurons [70]. In addition, support for retrograde vesicle-mediated transport has been attained by characterizing endosomes isolated from lumbar (L) 4 and L5 DRG neurons associated with the sciatic nerve [65]. Not only was NGF transmitted via axonal transport of early endosomes, the latter also contained its receptor, TrkA, as well as activated intracellular signaling proteins including ERK1/2, p38MAPK, and PI3K/Akt. Moreover, phosphorylated p38MAPK (p-p38) and the activated form of activating transcription factor 2 (ATF-2), which is stimulated by p-p38 and associates with DNA, either as a homodimer or after heterodimerizing with c-Jun, were also present in the early endosomes [65], suggesting that target gene transcription was occurring in relevant DRG.

By activating downstream target genes, ciliary neurotrophic factor (CNTF) as well as the glycoprotein 130 (gp130)-associated cytokines, leukemia inhibitory factor (LIF) and IL-6, allow for a greater number of growth processes to occur in the DRG (reviewed in [63]). Expanding briefly on the role of IL-6 in peripheral nerve injury-associated signal transduction, this particular cytokine binds its axonal membrane receptor and associates with the gp130 transmembrane protein, thereby initiating a cascade involving signal transducer and activator of transcription 3 (STAT3) [71, 72]. It has been demonstrated that chronic constriction of the sciatic nerve triggers IL-6 production and a resultant increase in the activation of STAT3 signaling within the nerve [72]. Axonal STAT3, which is activated at the injury site, acts as a transcription factor in sensory neurons [73], with activated STAT3 dimers traveling to the neuronal nucleus within the soma to promote the transcription of a specific repertoire of target genes [72, 74, 75]. Finally, negative injury signaling hinders the retrograde transport of trophic factors and serves as a negative regulator of neuronal growth, including, for example, via the transforming growth factor (TGF)-β/SMAD2/SMAD3 pathway (reviewed in [63]). The consequent outcome of peripheral axonal injury is a change in the expression of nociceptor-associated genes within the DRG, which play roles in inflammation, cell death, and nociception (reviewed in [76]).

### A case for exploring cell types other than nociceptors

In addition to action potential generation and retrograde signaling, there is significant evidence to implicate direct crosstalk between immune cell populations and the nervous system at the level of the DRG, as well as spinally, in response to a peripheral nerve injury. This is particularly relevant, given that the portion of a DRG that branches off at the T-junction to the soma, from axons of various distinct classes of nociceptive fibers, also contain satellite cells [77], as well as fibroblasts, macrophages, T and B lymphocytes, and endothelial and smooth muscle cells that represent their vascular component (reviewed in [78]). Each of these non-neuronal cells may have a significant influence on pain signaling.

A mechanistic approach commonly used in preclinical pain research is the assessment of nociceptor expression profiles at the single-cell RNA level in relevant DRG somata. A recent study employing such a strategy examined the molecular profile of primary afferents from naive male and female mice, as well as animals of each sex that had undergone a partial sciatic nerve ligation to mimic a state of neuropathic pain [79]. Lopes et al. first applied flow cytometry–based cell sorting to purify sensory neurons from dissociated DRG of non-injury-bearing naive rodents. This tactic provided the power to detect twofold changes in transcript levels via RNA sequencing (RNAseq) with a high degree of certainty. However, only a small repertoire of transcripts, the majority of which were associated with either the X or Y chromosomes, was differentially expressed between the sexes. The majority of these mRNAs have also been shown to be differentially expressed in human post-mortem [80] and neonatal mouse [81] brains. Surprisingly, using the same cell sorting approach to restrict RNAseq to sensory neurons, an almost identical set of transcripts was found to be differentially expressed in male and female ipsilateral L3 to L5 DRG (the region associated with the sciatic nerve) collected 8 days post-partial sciatic nerve ligation [79]. Importantly, a clear sex difference emerged only when peripheral immune cell infiltration into the DRG was examined. While a greater number of cell-sorted macrophages, monocytes, and neutrophils were present in DRG of both sexes following pain-inducing nerve impingement, more B cells were detected in males, and more T lymphocytes were detected in females [79]. These findings highlight the importance of separately examining neurons and other cell types, particularly cells associated with adaptive and innate immune responses, to systematically categorize the source of sexual dimorphisms in gene expression that contributes to sciatic nerve–associated neuropathic pain. This notion is supported by findings from previous RNAseq studies that were carried out in relevant preclinical models. These studies were based on examining the entire DRG cell population, demonstrating individual transcript differences in males (ipsilateral versus contralateral DRG) [82, 83] or in males compared with females [54] without providing cell-specific context. While Hu et al. did employ selective single-cell RNAseq, different types of DRG sensory neurons were manually picked based on cell diameter alone, without any further characterization to definitively isolate neurons from other resident cell types, including immune cells [84].

## Immune responses related to sciatic nerve injury: an overview

### Relevant immune cell types and cytokines

**Fig. 1:**
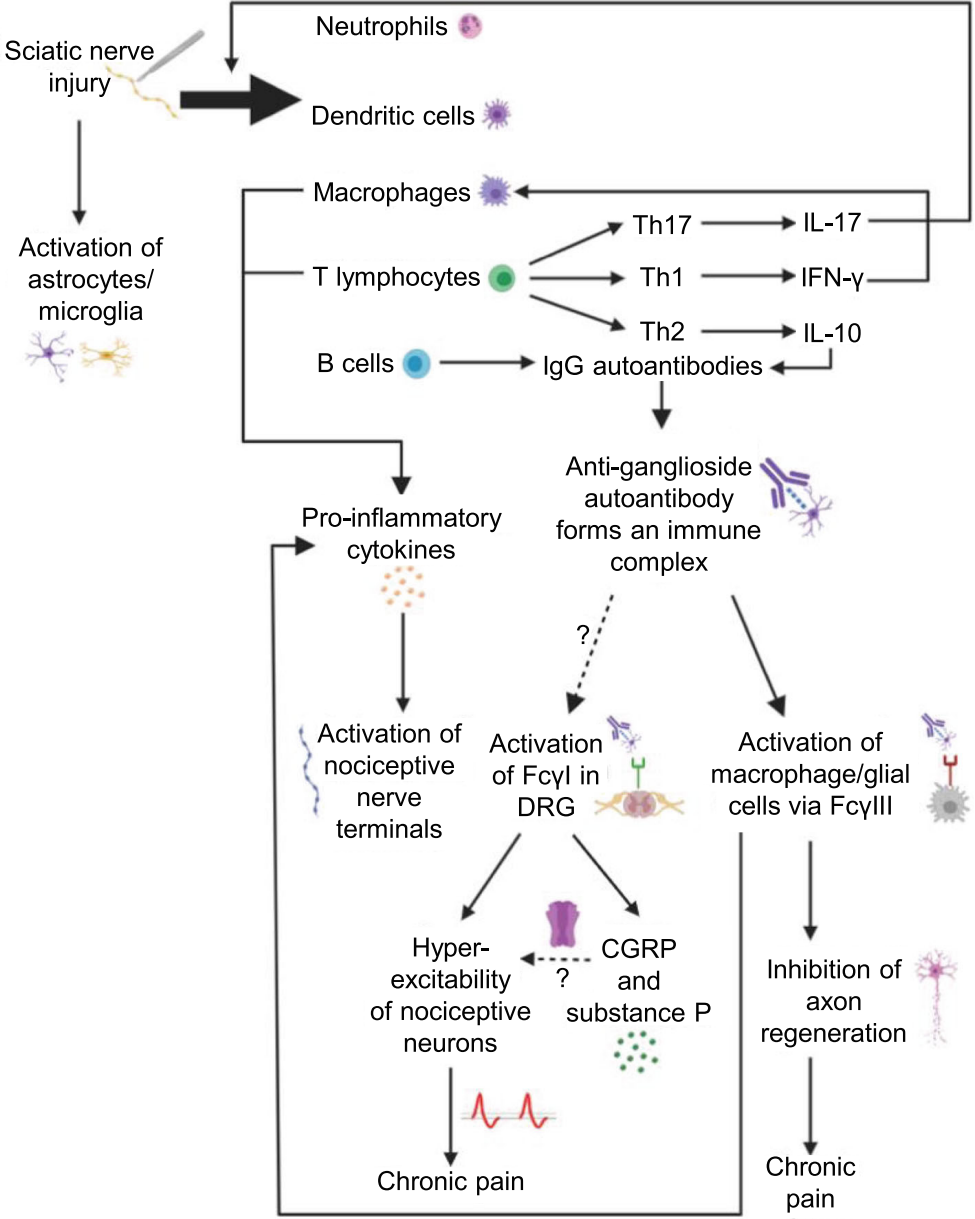
Neuro(auto)immune response to sciatic nerve injury. Using the sciatic nerve as an example, this figure provides a snapshot overview of the events that may underlie a neuroimmune response, which may involve aspects of aberrant autoimmune interactions, to a peripheral nerve injury. Following nerve injury, depolarizing voltages as well as positive and negative injury signaling mechanisms transduce information about the injury to the DRG, allowing for the coordination of a regenerative response. From the DRG, signals are transmitted from the periphery to the spinal cord by cytokines, nucleotides, and chemokines, resulting in microglial and astrocytic activation in the dorsal spinal horn. Ultimately, these signals are transduced to centers in the brain for further processing. Various immune populations are recruited to the site of sciatic nerve injury and the ipsilateral DRG. The factors influencing immune cell recruitment are complex and include toll-like receptors, Schwann cells, and other immune populations, cytokines, and chemokines. The activity of these immune cells is dynamic, and in many instances, may not be localized to a singular region. In the context of sciatic nerve injury, there is evidence of neutrophil, dendritic cell, macrophage, and T lymphocyte presence at the site of injury and ipsilateral DRG. The latter two immune cell types produce pro-inflammatory cytokines such as TNF-α, which can activate nociceptive nerve terminals. Th1 lymphocytes produce IFN-γ, which regulates macrophage activity and is detected at the sciatic nerve after injury. IL-17, produced by Th17 lymphocytes, plays a role in mediating infiltration of T lymphocytes to the site of injury as well as activation of microglia and astrocytes. B lymphocytes are also recruited to the site of injury and can produce pathological IgG autoantibodies, including those that form an immune complex when bound to gangliosides on neuronal and axonal cell surfaces. Autoantibody production may be promoted by IL-10, which is produced by Th2 lymphocytes and is detected at the sciatic nerve after injury. Anti-ganglioside autoantibody immune complexes interact with FcγRIII on macrophages and glial cells at the site of injury, leading to pro-inflammatory cytokine production and inhibition of axon regeneration, thus likely resulting in chronic pain. These immune complexes may interact with FcγRI in the DRG; this interaction generates excitable activity in nociceptive neurons and may result in the secretion of CGRP and substance P in the DRG. Both CGRP and substance P are involved in signal transduction and the latter neuropeptide may promote long-term potentiation of excitable currents generated via the NMDAR. Prolonged hyper-excitability of nociceptive neurons may eventually result in chronic pain

As mentioned earlier in this review, immune cells secrete factors that are able to directly influence neuronal activity, phosphorylating ligand-gated channels, modulating the activity of voltage-gated ion channels, and increasing intracellular calcium levels through activation of various receptors including neuronally expressed cytokine receptors (reviewed in [41, 85]). The end result is an alteration of membrane properties and greater action potential generation, along with signal propagation to higher-order centers, for heightened pain sensation in response to injury or insult. In addition, various immune cells are also present within the DRG and the spinal cord, potentially contributing to sexually dimorphic signaling that may contribute to the chronification of pain. A general overview of immune activity associated with neuropathic pain that may be induced by a sciatic nerve injury is depicted in Fig. 1. A brief functional summary of several key immune cell types and cytokines is also provided here to establish their context in pain-evoking neuroinflammatory processes.

Dendritic cells are specialized antigen-presenting cells that stimulate naive T cells (reviewed in [86]), with the latter, in their differentiated form, in turn promoting B cell differentiation and providing support in responses to certain antigens (reviewed in [87]). Macrophages engulf and destroy pathogenic substances and infected cells (reviewed in [88, 89]), also playing a role in the activation of B and T lymphocytes (reviewed in [88, 89]). B lymphocytes are a distinct adaptive immune cell population that protects against specific antigens by secreting antibodies and pro-inflammatory cytokines such as IL-10 (reviewed in [90]). Broadly, CD4^+^ T helper (Th) lymphocytes produce a repertoire of specific cytokines that support adaptive immune responses—for example, by contributing to the activation of cytotoxic CD8^+^ T cells and macrophages, as well as the maturation of B cells into plasma or memory B cells, B lymphocyte-driven antibody production, as well as immune tolerance, or suppression thereof (reviewed in [91–93]). Neutrophils are another versatile cell type that facilitate microbial destruction through a variety of mechanisms, also mediating inflammatory processes [94].

Many of these immune cells of peripheral origin secrete repertoires of ILs, as well as interferon gamma (IFN-γ) and tumor necrosis factor alpha (TNF-α) [95]. Some of these factors are able to directly activate nociceptive nerve terminals, leading to sensitization, aberrant action potential generation, and neuropathic pain (reviewed in [95, 96]). For example, TNF-α is able to directly activate TNF-α receptors on peripheral nerve terminals, with this interaction shown to amplify hyperalgesia [97, 98]. The activity of immune cells, however, may not be localized to a singular region, and aberrant action potential generation that occurs in response to a sciatic nerve injury, which results in a hyper-excitable state, does not have to occur at sensory nerve endings. Rather, these action potentials can occur along the damaged axon itself [99, 100]. Certain cytokines also extensively influence the activity of other immune cell types. For instance, IFN-γ, most notably produced by T lymphocytes and natural killer cells, promotes the development and activation of CD4^+^ Th 1 (Th1) cells and stimulates the expression of the major histocompatibility complex class II (MHC-II), thereby promoting macrophage activity and inducing the secretion of other cytokines (reviewed in [101]). In addition, IFN-γ receptor signaling has been shown to mediate the activation of microglia in the CNS to drive neuropathic pain [102]. Another relevant subset of Th cells, Th17 lymphocytes produce the cytokine IL-17. The primary function of IL-17 is to provide protective immunity against pathogens by contributing to neutrophil activation, but it also participates in pro-inflammatory and pathological autoimmune processes (reviewed in [103, 104]). It should be noted that, in addition to these adaptive Th17 cells, IL-17 is also produced by γδ T cells [105], which play a role in innate immune responses (reviewed in [106]).

### At the site of the peripherally injured sciatic nerve and associated DRG

As a first step in the peripheral nerve injury–induced inflammatory process, immune cells are recruited to the site of injury as well as to the associated DRG. This is facilitated through activation of toll-like receptors (TLR), which are expressed on several immune cell types (reviewed in [42]). TLRs respond to an accumulation of damage-associated cellular debris, triggering nuclear factor-κB (NF-κB) and subsequent transcriptionally mediated cytokine synthesis (reviewed in [42, 107]). For example, TLR4, which is primarily known to respond to lipopolysaccharide (LPS), is expressed on B and T lymphocytes, dendritic cells, and neutrophils, as well as astrocytes and microglia [108, 109]. In mice with a partially ligated sciatic nerve that experience mechanical and thermal hypersensitivity in the injury-associated limb, dendritic cells, macrophages, neutrophils, and lymphocytes infiltrate the area around the impinged nerve and the corresponding ipsilateral DRG [110]. Mast cells have also been identified as a component of the immune response associated with nociception following sciatic nerve ligation, particularly by influencing the recruitment of neutrophils and monocytes to the injured nerve [111]. Moreover, a significant increase in the expression of IFN-γ, IL-10, and the IFN-γ/IL-10 ratio has been observed in rats 1 week after inducing a sciatic nerve injury [112].

Schwann cells, which, in the PNS, comprise the myelin sheath by wrapping around the neuronal axon, also respond to cellular debris and release neurotrophic factors, cytokines, and chemokines to attract phagocytes to the site of injury [113]. Schwann cell–secreted TNF-α activates matrix metallopeptidase 9 (MMP-9), which facilitates macrophage migration to the injured site ( [114]; reviewed in [107]). Neurogenic inflammation, which involves the secretion of primary afferent neuronal signals and neuropeptides [115], plays an integral role in neutrophil recruitment (reviewed in [107]). In particular, the neuropeptides substance P and CGRP are significantly involved in pain transduction [116–118]. With regard to immune cell recruitment, the cooperative activity of TNF-α and IL-17 has also been shown to facilitate neutrophil infiltration into the damaged area [119]. IL-17 also interacts directly with its receptor at nociceptive nerve terminals (reviewed in [120]. In IL-17 knockout mice with a peripheral nerve injury, decreased mechanical hypersensitivity has been shown to be accompanied by decreased T cell and macrophage infiltration to the injured sciatic nerves and the L3-5 DRG [121]. In addition, local administration of IL-17 is associated with increased numbers of activated dendritic cells and infiltrating neutrophils at the site of the injected hind paws, and increased neutrophil infiltration in the injected sciatic nerves [121]. While it is known that CD8^+^ cytotoxic T cells are recruited to peripherally injured nerves, responding to elevated antigen-presenting MHC class I molecules, the precise mechanism underlying this process remains unclear ( [122, 123]; reviewed in [124]). It was recently shown that Treg cells also play a role at the site of a peripheral nerve injury, counteracting neuropathic pain by inhibiting Th1 cell-mediated responses [125], and it has been reported that there is a disrupted Th17/Treg balance in patients with chronic low back pain [126].

Chemokine (C-C motif) ligand 2 (CCL2), also known as monocyte chemoattractant protein 1 (MCP-1), produced by primary afferent neurons in the spinal dorsal horn, is released in synaptic vesicles following peripheral nerve injury [127]. This chemokine has been shown to promote the recruitment of monocytes, neutrophils, T cells, and dendritic cells to the injury site, resulting in significant inflammation [128–130]. With regard to the DRG, the factors and exact mechanisms influencing immune cell recruitment to this area of the injured nerve also remain to be defined. However, chemokine expression (such as CCL2/MCP-1 as well as CCL3/MIP-1) may play a significant role, particularly in the context of macrophage infiltration, which appears to be mediated by the toll-like receptor 2 (TLR2) present in the DRG [131].

Once recruited to the site of neuronal injury, the immune cell milieu is able to contribute to nociceptor signaling by triggering aberrant action potential generation in a localized manner. Other, possibly concurrent processes, which have yet to be clearly defined, may contribute to nerve injury signaling to the DRG soma. The primary means by which secreted cytokines cause nociception is by modulating the activity of voltage-gated sodium channels (Na_v_1.7, Na_v_1.8, and Na_v_1.9) and TRP ion channels [TRPV1 and TRP ankyrin 1 (TRPA1)] (reviewed in [41]). Interestingly, it has been shown that much of the CCL2 released in the dorsal horn of the spinal cord of nerve-injured rodents originates from TRPV1-positive nociceptive fibers [127], suggesting the presence of feedback loops that perpetuate neuroimmune interactions. Ultimately, activation of these channels via neuroimmune interactions causes increased action potential generation and firing of a nociceptive neuron, which has been extensively reviewed by others [41, 132]. As an example, TNF-α activates the TNF-α receptor 1 (TNFR1) to promote Na_v_1.9 phosphorylation, while IL-6, by activating the gp130 channel, increases the expression of TRPV1 and TRPA1 (reviewed in [41]). Application of IL-17A into the DRG of a rat arthritis model reduced the threshold required to stimulate an action potential, also increasing the number of action potentials generated [133], providing additional insight into the complex nature of this cytokine’s contribution to pain. Overall, cytokines (such as TNF-α, IL-1β, IL-6, and IL-10) and chemokines (such as CCL2/MCP-1), along with sodium and TRP channels, increase the primary afferent signals that are sent via the DRG into the spinal dorsal horn (reviewed in [107]). Of note, while TNF and IL-1β contribute to neuropathic pain in mice with a sciatic nerve injury, these cytokines are also required for functional recovery [48]. Indeed, recovery was impaired in TNF, IL-1β, and TNF/IL-1β knockout mice [48]. Taken together, these findings suggest that homeostatic dysregulation of cytokine production, rather than their mere presence, contributes to neuropathic pain occurring in response to a sciatic nerve injury.

### From the DRG to the spinal cord

The next step in ascending nociceptive signaling involves the transduction of the signal from the periphery into the spinal cord. Microglia, which are considered the “resident macrophages of the CNS,” are activated by the described sensory afferent-derived injury signals. These may include pro-inflammatory cytokines, nucleotides, and chemokines that are released through post-synaptic surface receptors as well by other immune cells, which is accompanied by the activation of pattern recognition receptors such as TLR2 (reviewed in [134]). A significant increase in microglial and astrocytic activation occurs in the ipsilateral spinal dorsal horn of nerve-injured animals [110]. In turn, microglia secrete pro-inflammatory cytokines, reactive oxygen species, and brain-derived neurotrophic factor (BDNF) into the spinal cord to communicate with other components of the neuronal network and other immune cells (reviewed in [134]).

Accumulating evidence supports that spinal CD4^+^ T cell–dependent responses contribute to the maintenance of neuropathic pain. The infiltration of lymphocytes into the dorsal spinal cord and their subsequent release of cytokines have been shown to play key roles in “neuropathic pain–like hypersensitivity” in an adult rodent model of peripheral nerve injury, which does not occur in neonates [135]. Furthermore, Sun et al. showed that not only is CD4^+^ T cell infiltration into the spinal cord increased in a rat model of peripheral nerve injury, significant upregulation of IL-17, as well as enhanced mRNA levels of IL-1β and IL-6 and astrocytic proliferation also occur in the spinal dorsal horn of injured rats compared with sham animals [136]. In IL-17 knockout mice bearing a peripheral sciatic nerve injury, mechanical hypersensitivity was shown to be significantly decreased, concomitant with decreased astrocytic and microglial activation in the L3–L5 dorsal and ventral horns of the spinal cord [121]. The functional role of T cells that cross the blood-spinal cord barrier has been emphasized by work in T cell–deficient rodents, which exhibit reduced neuropathic pain–associated tactile allodynia [135, 137, 138]. This latter pro-nociceptive response could be reversed by adoptive transfer of splenic CD4^+^ T cells derived from nerve-injured animals [137, 138]. In addition, in mice lacking IFN-γ receptors, peripheral nerve injury–induced tactile allodynia and activation of spinal microglia have been shown to be attenuated [102, 135].

#### More on microglia

In a normal physiological state, microglia are “resting” yet dynamic, continuously scanning their environment for any changes that could alter homeostasis [139, 140]. Upon exposure to certain signals, microglia are activated to perform innate immune functions [140]. In this latter state, microglia drive synaptic alterations within the dorsal horn of the spinal cord, representing a key pro-nociceptive event (reviewed in [141]). It is thought that microglia that fail to return to a resting state contribute to persistent neuropathic pain (reviewed in [142]).

The finding that the purinergic receptor 4 (P2X4), a highly sensitive ligand-gated ion channel, is expressed by microglia highlighted their importance in mediating peripherally induced nociceptive hypersensitivity in rodent models of pain [143]. In response to nerve injury, transcriptional upregulation of microglial P2X4 expression was found to be linked with specific ATP-mediated signaling events associated with dysregulated chloride transport (reviewed in [144]). Pharmacologically inhibiting or genetically blocking P2X4 in the spinal cord of rodents abrogates nociceptive hypersensitivity [143, 145], although it is important to note that this would affect both microglia as well as central neurons, as P2X4 expression is not microglially restricted [146]. It is also important to note that this particular pro-nociceptive signaling pathway was characterized in male rodents [143, 147–150]. Similarly, innate immune response-associated TLR4, the CNS expression of which is primarily restricted to microglia, has been shown to only contribute to hypersensitivity in male mice [151, 152].

It has been suggested that specific microglial activities, which may “exhibit a spectrum of distinct functional states,” may be restricted in the CNS, occurring in an extremely localized manner [153]. In support of this notion, it was recently reported that microglia are differentially activated in the anterior and posterior horn of the spinal cord after chronic constriction injury in male rats [154]. Nishihara et al. demonstrated that anterior microglial activation may result in synaptic stripping, while activated microglia in the posterior horn may be engaged in phagocytic myelin removal, indicating that differences in microglial activation modes may lead to distinct symptoms that arise in response to peripheral sciatic nerve constriction [154]. It will be of considerable interest to examine whether sex differences are associated with these distinct functions. Of note, Lopes et al. isolated a 98% pure population of murine microglia from male and female ipsilateral lumbar spinal cords 8 days post-nerve ligation. Microglial proliferation rates were comparable between the sexes, and a quantitative PCR–based evaluation of the expression of numerous transcripts associated with reactive microglia, nociception, and adaptive immunity at the spinal cord level showed no significant sex differences [79]. While it is now beginning to be accepted that microglia may not be the primary drivers of persistent nociceptive signaling in females [155], evaluating the roles of microglia in males and females with a similar nerve injury at the single-cell, or clonal, level may provide specific context-dependent insights. Males and females also exhibit differences in immune system activity involving specific T cell populations, both prior to and following a pain-inducing peripheral nerve injury, and these cells are now thought to play a key sexually dimorphic role in the onset of pain and its chronification (reviewed in [156]).

### Evidence from patients

There is a growing body of evidence to suggest the involvement of inflammatory processes in chronic sciatic conditions, as well as autoimmune pathologies, in humans. IL-1β, IL-10, TNF-α, and IL-17 have been detected in human patients with sciatica and could be considered potential serum, biopsy, or cerebrospinal fluid biomarkers (reviewed in [157]). Consistently, Andrade et al. detected IL-10, as well as TNF-α and IL-1β, in the cerebrospinal fluid of patients with thoracic disc herniation, with IL-10 negatively, and TNF-α positively, associated with high pain scores [158]. Although it promotes regenerative activities, IL-10 is also involved in antibody production and has been implicated in several painful autoimmune diseases (reviewed in [159]), including the production of autoantibodies in systemic lupus erythematosus [160]. In a recent systematic review focused on underlying pathogenic mechanisms in sciatica, IL-1β, IL-6, TNF-α, CCL2, IL-17, and IL-21 were identified as potential biomarkers, with Jungen et al. reporting a strong positive correlation in longitudinal studies between IL-21 and pain [157]. Of relevant interest, IL-21 is synthesized by Th17 cells, driving their production of IL-17 [161], and IL-17 has also been shown to contribute to inflammatory autoimmune pathologies such as multiple sclerosis and rheumatoid arthritis (reviewed in [103, 162]).

## Autoimmune pathology

Autoimmunity represents a pathological process whereby antibodies target self-antigens, and in certain contexts, contribute to neuropathic pain. There is significant evidence to suggest that autoimmune processes are painful (reviewed in [163]). In many cases, autoimmune disorders involve B cell–produced immunoglobin G (IgG) antibodies directed against specific self-antigens. Interactions between IgG and the Fc gamma receptor (FcγR) play a role in inflammatory autoimmune diseases (reviewed in [164, 165]), with IgG acting as the major ligand that links humoral and cellular immune mechanisms. It mediates both pro- and anti-inflammatory effects following immune complex formation and engagement with different activating or inhibitory FcγRs, which are divided into three main classes: FcγR type I (FcγRI; CD64), FcγRII (CD32), and FcγRIII (CD16). Dendritic cells may worsen the damage by further promoting antibody production (reviewed in [166]).

### Fc gamma receptors

FcγRs are present on phagocytes (macrophages and monocytes), granulocytes (eosinophils and neutrophils), and lymphocytes (B cells and natural killer cells) [167, 168], facilitating the binding of these cells to the Fc region on antibodies that have become attached to the surface of pathogens, infected cells, or self-antigens. This in turn results in activation of the FcR-expressing cell, and immune activity. Indeed, macrophage activation requires a balance between immune complexes that bear FcRs [169]. Many immunological processes are triggered by FcγR cross-linking, such as setting the threshold for B cell activation, antigen presentation, antibody-dependent cellular cytotoxicity, degranulation, leukocyte recruitment, phagocytosis, and the release of pro-inflammatory mediators [165, 170].

A recent transcriptome analysis of the immune system demonstrated that expression of specific FcγRs is elevated in female rats [171]. The finding that B lymphocytes and IgG are present at the site of a sciatic nerve injury in a relevant animal model provides support to the notion that an underlying autoimmune component influences the outcome of a peripheral nerve injury [110, 172]. Differences in FcγR expression between the sexes may contribute to the sexually dimorphic prevalence of certain autoimmune disorders, as well as the higher incidence of unresolved persistent pain, in women.

#### FcγRI

It has been shown that immune complexes interact with the FcγR type I (FcγRI), leading to excitation of certain nociceptive neurons in rat DRG [173]. The majority of very small-diameter nociceptors that expressed FcγRI co-expressed the nociceptive ion channel TRPV1 [173], which also regulates the activation and pro-inflammatory properties of CD4^+^ T lymphocytes [174] and may be differentially expressed in males and females in response to sex hormones [175–178]. While neither antibody (IgG) nor antigen alone generated an increase in intracellular calcium levels, the entire immune complex was able to elicit this effect, which was abolished by the removal of the IgG Fc portion or application of an anti-FcγRI antibody [173]. In addition, either depleting extracellular calcium levels or intracellular calcium stores prevented the immune complex–induced calcium response [173]. By eliciting a prolonged hyper-excited state, immune complex-FcγRI binding may contribute to persistent neuropathic pain by promoting nociceptor sensitization [96, 173].

Activation of FcγRI in the DRG also leads to the secretion of substance P, which may act on its own receptors within DRG [179, 180]. Furthermore, substance P can induce long-term potentiation of the excitable current generated via NMDAR in the dorsal horn of the rat spinal cord [181]. Previous studies have shown that substance P and CGRP enhance vasodilation and contribute to nociception (reviewed in [107]). Interestingly, significant upregulated expression of both of these neuropeptides has been reported in relevant rat DRG with a peripheral sciatic nerve injury [182], indicating that hypersensitivity following nerve damage may involve immune complex-FcγRI-mediated neuropeptide secretion which, in turn, augments neuronal excitation evoked by this interaction. It is important to reiterate here that neuropeptides, although often co-released with various neurotransmitters, undergo significantly different processing within a nerve. Unlike neurotransmitters, which are synthesized in nerve terminals and are then taken up into the presynaptic nerve ending following their initia release, neuropeptides are synthesized in the neural soma within DRG, transported to the synapse, and, once released, are then metabolized. This requires new synthesis as well as axonal transport for their continued action. Of additional note, and adding to the existing complexity of neuroimmune interactions, immune cells themselves, including dendritic cells, lymphocytes, macrophages, mast cells, and monocytes, also produce neuropeptides in response to antigens and inflammation, which then act either on nerves in a paracrine manner, or interact with specific receptors that are expressed on immune cells in an autocrine fashion (reviewed in [183, 184]). Upon their release, CGRP and substance P are able to directly stimulate or inhibit T cell activation and also produce indirect effects by influencing the recruitment and activation of dendritic cells [184]. Evidence suggests that chemokine expression within the DRG, which is associated with excitatory signals and pain, promotes the secretion of substance P [185], presenting chemokines as another possible mechanism by which injury signals are communicated to the DRG.

#### FcγRIII

FcγRIII exists as two highly homologous isoforms: FcγRIIIA (CD16A) is expressed by mast cells, macrophages, natural killer cells, and neutrophils, while the expression of FcγRIIIB (CD16B) is restricted to neutrophils [186, 187]. Together, these receptor isoforms stimulate degranulation, phagocytosis, and oxidative burst, allowing neutrophils to clear opsonized pathogens [186]. In healthy individuals, CD16 cross-linking by immune complexes induces antibody-dependent cellular cytotoxicity. Downregulation of CD16 represents a possible means to moderate natural killer cell responses and to maintain immune homeostasis in both T cell- and antibody-dependent signaling pathways [188]. A FcγRIIIA allelic variant that enhances IgG1 affinity and natural killer cell activation is among one of the best-studied human FcR polymorphisms [189]. B cells play a central pathogenic role in autoimmune systemic lupus erythematosus, and the monoclonal antibody–based therapeutic agent rituximab depletes B cells in patients, with its efficacy highly dependent on the FcγRIIIA genotype [189].

Immune complex interactions with FcγRIII may lead to persistent pain following sciatic nerve injury through a mechanism involving gangliosides, molecules with a glycosphingolipid, sialic acid, and saccharide component. Autoantibodies against the neuronal and axonal cell surface gangliosides GD1a and GT1b have been studied from patients with Guillain-Barré syndrome, an autoimmune neurological disease [190] that is commonly associated with significant pain [191]. These same autoantibodies interacted with gangliosides on injured axons in mice with a sciatic nerve crush injury [190]. The resulting immune complexes also activated FcγRIIIs on glial cells and monocyte-derived macrophages at the sciatic nerve, resulting in an inhibition of axon regeneration [190]. Immune complex-FcγR binding on macrophages is known to trigger the release of pro-inflammatory cytokines, and may thereby contribute to hyper-excitability (reviewed in [165]). Furthermore, while it was shown that immune complexes involving autoantibodies against GD1a and GT1b had the highest binding affinity for FcγRI, this receptor, unlike FcγRIII, did not appear to play a role in the inhibition of axon regeneration [190]. Additional research needs to be carried out to examine whether immune complexes formed by these specific autoantibodies also contribute to hyper-excitability via FcγRIII activation in the DRG following impingement of the sciatic nerve.

Based on the composition of gangliosides, an enzyme-linked immunosorbent assay was developed to evaluate autoantibodies targeted against glycosphingolipids in patients with sciatica [192]. Elevated levels of antibodies against glycosphingolipids occurred in 71% of patients with acute sciatica and 61% of patients with chronic sciatica upon 4-year follow-up [192]. These findings support the possibility that an autoimmune mechanism involving FcγRs may contribute to chronic pain stemming from an injury to the periphery, with potential underlying sex differences in pain resolution.

### Autoantibodies: the example of complex regional pain syndrome

While separate conditions, persistent lower-limb pain stemming from post-surgical sciatica (pain that develops, for example, from low back surgery) and lower limb-associated complex regional pain syndrome share certain mechanisms, including inflammation, dysregulated neuroimmune cross-talk, and central neuroplasticity [193]. Most patients diagnosed with complex regional pain syndrome, a post-traumatic neuralgia that generally affects a single limb without evident tissue damage [194, 195], undergo spontaneous recovery. However, up to 20% develop severe persistent pain that may last a lifetime [196, 197]. Its severity is independent of the initiating trauma, which in many cases takes the form of seemingly inconsequential insults [198, 199]. Studies have demonstrated that administration of IgG, derived from patients with complex regional pain syndrome that continued longer than a year, to healthy mice reduced their spontaneous rearing behavior [200]. IgG transfer coupled with an experimental insult in the form of a paw incision resulted in transient post-surgical swelling and mechanical hypersensitivity in the affected limb [201]. Other immunoglobulins were inactive, based on the finding that the transfer of IgG-depleted serum did not produce any effects [202].

In a recent study, Cuhadar et al. identified peripheral nociceptor sensitization as a major mechanism by which autoantibodies may produce pain in complex regional pain syndrome [203]. Female mice were subjected to a minor experimental insult, concomitant with the administration of patient-derived IgG, resulting in persisting mechanical and thermal sensory changes. Furthermore, the degree of transferred hyperalgesia was correlated with the dose of IgG and donor patient pain scores, as reduced IgG-mediated nociceptive responses were recorded in animals receiving IgG transferred from patients reporting only moderate levels of pain [203]. Importantly, in ex vivo cutaneous nerve preparations, the spontaneous and evoked action potential discharge rates were increased, demonstrating that patient IgG autoantibodies generated nociceptor hyper-excitability [203].

Of note, complex regional pain syndrome has recently been shown to involve both the expansion and activation of distinct subsets of memory T cells [204, 205]. Furthermore, the profile of tissue-resident cutaneous T cells in the limb affected by complex regional pain syndrome is also altered compared with non-affected areas in a manner suggestive of a Th2 cell bias [206]. Elevated circulating levels of the soluble IL-2 receptor have been detected in patients relative to healthy controls, suggesting that a T cell-mediated inflammatory process could be a key component [207]. The triggering of neoantigen production provides a possible link between the initial trauma and the resulting autoantibody-mediated pathological outcome. A compromised vascular-neural barrier around the affected area could lead to plasma extravasation [208], allowing IgG to gain access to the damaged site to interact with these antigens. Immune therapies, such as B cell ablation or plasmapheresis, may represent a means to reduce autoantibody titer, which could potentially also be applied to patients with persistent sciatic nerve pain.

## The sex chromosomes

Sex chromosome-associated genes exhibit disparate expression profiles that arise independent of an individual’s hormonal status and therefore represent a significant driver underlying the differences between males and females under normal and pathological conditions of the nervous and immune systems (recently reviewed in [209–213]). This is particularly relevant, given that sex-specific actions of X and Y genes in nociception and analgesia have been shown to occur by the day of birth in mice [214]. To examine the importance of sex chromosomes, the “four core genotypes” mouse model has been developed to study the contribution of sex chromosome complement to various physiological networks, independent of gonadal sex [215]. Studies based on this model have demonstrated XX versus XY differences in behavior, disease susceptibility, and gene expression that are not mediated by gonadal hormones, which may instead be associated with the dose of X chromosome genes or parental epigenetic imprinting events. Further identification and characterization of genes on the sex chromosomes remains an active area in the field of pain research and could contribute to improved sex-specific therapies.

### The X chromosome

The X chromosome represents approximately 2.5% of the total DNA within each male mammalian cell, with this dosage doubled in females. During the early stages of female embryogenesis, one of the two X chromosomes randomly undergoes permanent somatic cell X-inactivation to ensure that females resemble males in maintaining only one functional copy of this chromosome per somatic cell. Silencing of the X chromosome occurs via two main mechanisms: epigenetic changes that include chromatin modification [216] and the coating of one X chromosome by the X-inactive specific transcript (XIST), a long non-coding RNA [217–219]. The latter requires the Ying Yang 1 (YY1) protein, which activates XIST [220] and secures it to the X chromosome [221]. Of note, 10 to 15% of genes that localize to the X chromosome escape X inactivation, resulting in their bi-allelic expression and skewed transcript levels in females [222]. Escape from X inactivation occurs mainly to genes within the pseudoautosomal region at the tip of the short arm of the X chromosome, representing non-recombining sequences. Many of these genes have been associated with a pathological state, including major psychiatric disorder, systemic lupus erythematosus, Rett syndrome, and thyroid autoimmunity [223–225]. It should be noted that, in addition to genes located on the X chromosome, there may also be somatic genes associated with nociceptive sensitivity that may be differentially expressed due to altered expression of regulatory factors that are X-linked, as reported in autism spectrum disorders (reviewed in [226]).

Numerous sexually dimorphic differences in disease susceptibility may potentially be attributed to changes in the expression of genes associated with an escape from X inactivation. In support of this notion, sex chromosome abnormalities contribute to brain disorders [227], and X chromosome inactivation is associated with neural development, function, and disease [228]. Murine YY1 plays a role in inflammatory pain and morphine analgesia, as assessed using a Cre/lox strategy to ablate its expression in Nav1.8-positive DRG neurons [229]. In addition, human YY1 may be relevant to pain, as a microarray gene-expression profile of synovial membrane samples revealed that this gene was crucial in the regulatory network of rheumatoid arthritis [230]. Interestingly, the expression of YY1 appears to be important for proper *XIST* localization [231], which may be required for silencing of the X chromosome. Wang et al. showed that in women with systemic lupus erythematosus, *XIST* is dispersed in naive lymphocytes, resulting in gene escape from X chromosome inactivation [231].

Lower back pain and disc herniation/sciatica are common features of a motor vehicle collision [232, 233]. Interestingly, the majority of individuals who develop chronic musculoskeletal pain [234, 235] and/or symptoms of post-traumatic stress [236] following a motor vehicle collision are women, with *XIST* found to be significantly dysregulated [225]. A recent study by Yu et al. reported that, during the early stages following a collision, 40 genes originating from the X chromosome were differentially expressed in women who later developed chronic musculoskeletal pain and/or signs of post-traumatic stress compared with those who recovered [237]. In contrast, the repertoire of 25 X chromosome genes found to be differentially expressed in men was distinct from the set identified in women. Unlike in men, two well-defined clusters categorized by pathway analysis were enriched for genes known to escape X chromosome inactivation. These clusters were based on upregulated expression of genes associated with the eukaryotic initiation factor 2 (EIF2) pathway or IL-2 signaling [237].

#### EIF2 and IL-2

Ubiquitously expressed, EIF2 is required for translation initiation by mediating the GTP-dependent binding of methionine-charged initiator tRNA to the ribosome. As a heterotrimer, it is comprised of three subunits, alpha (subunit 1, EIF2S1), beta (subunit 2, EIF2S2), and gamma (subunit 3, EIF2S3). EIF2 plays a role in cellular stress responses [238–240] and has also been associated with learning and neuroplasticity [241–243]. These latter two processes have been implicated in altering the function of the PNS and CNS during pain chronification and its resolution [51, 244].

Produced by activated CD4^+^ and CD8^+^ T cells, IL-2 mediates immune tolerance by directly affecting T lymphocytes [245]. Its expression and secretion are tightly regulated, with IL-2 functioning as part of positive and negative feedback loops in mounting and dampening immune responses, respectively. In the thymus, IL-2 promotes the differentiation of immature T cells into T regulatory (Treg) cells. The latter suppress T cell populations that are otherwise primed to “attack” healthy tissue, thereby preventing autoimmunity. In concert with other polarizing cytokines, IL-2 stimulates naive CD4^+^ T cell differentiation into Th1 and Th2 lymphocytes as well as their expansion, and blocks Th17 differentiation while also being able to expand this latter cell type [246]. Furthermore, IL-2 plays a key role in sustained cell-mediated immunity during the development of immunologic memory, which depends on the expansion of antigen-selected T cell clones [245, 247]. Importantly, IL-2 has been linked to the development of persistent pain [248, 249], identified as a potential pain biomarker in patients with sciatica [157], and associated with post-traumatic stress [250, 251].

#### SH2D1A, CD40LG, and EIF2S3

The majority of individual genes identified in non-recovering women in the collision study were associated with immune function and neuronal or cognitive activities [252, 253]. The transcript most significantly associated with pain and post-traumatic stress was X-linked *SH2D1A* (SH2 domain–containing protein 1A), which plays a role in stimulating T and B lymphocytes [254, 255] and mediating cytokine production [256]. *CD40LG*, another key X-linked transcript, is expressed on the surface of T cells and serves to regulate B cell function. In T cells of women with systemic lupus erythematosus, *CD40LG* has been shown to be demethylated on the inactive X chromosome [257], and its allelic variants are associated with rheumatoid arthritis [258]. In addition, *EIF2S3* mRNA levels were also associated with pain and post-traumatic stress [237]. EIF2S3 plays a direct role in synaptic plasticity and cognitive impairment [259, 260], as well as in EIF2-controlled thermal nociceptive responses [261].

#### KDM6A/UTX

In addition to *XIST*, epigenetic gene modifications also play a key role in X chromosome inactivation. A recent study examining the mRNA profile of CD4^+^ T cells found that the epigenetic modifier *KDM6A/UTX* (lysine-specific demethylase 6A), an X-linked member of the H3K27me3-specific demethylase subfamily, was expressed at a higher level in women than in men [262]. The authors postulated that sexually dimorphic expression of *KDM6A* in immune cells could provide insights into why more women than men generally develop autoimmune diseases. Upon knockout of *Kdm6a* in a classic mouse model of multiple sclerosis (CD4^+^ T cell–mediated experimental autoimmune encephalomyelitis), reduced inflammation and a reduction of spinal cord damage to neuronal axons were observed compared with wild-type counterparts [262]. Global transcriptome analysis in CD4^+^ T lymphocytes isolated from these knockout mice revealed that specific pathways associated with Th1 and Th2 cell activation were upregulated [262], and it was therefore suggested that modulating the activity *Kdm6a* in T lymphocytes could be a potential targeted therapeutic approach to treat multiple sclerosis and other autoimmune diseases in which these cells play a role.

Interestingly, metformin was recently shown to alter the activity of KDM6A/UTX [263]. Structural studies revealed that metformin could potentially occupy the catalytic pocket of this particular target via the same residues that are involved in H3K27me3 binding and demethylation [263]. Indeed, oral administration of pharmacological doses of metformin significantly increased global levels of H3K27me3 in murine liver and tumor tissues [263]. This study also showed that oral metformin, in combination with standard therapy, resulted in an increase in the level of circulating H3K27me3 in non-diabetic breast cancer patients [263]. Interestingly, metformin may have relevance in treating persistent pain. In male rats that underwent a complete laminectomy of the T9 vertebra followed by a spinal cord contusion injury, treatment with metformin decreased mechanical and thermal hypersensitivity, improved locomotor activity, and significantly lowered IL-1β and TNF-α levels in spinal cord specimens [264]. However, while metformin was recently shown to reverse nociceptive behavior in male mice using the spared nerve injury model, no effects were observed in female counterparts [265]. In this latter study, metformin administration in males decreased microglial activation in the spinal dorsal horn, and while robust microglial activation occurred in female injured mice, no parallel treatment-induced decrease occurred [265]. It will be of interest to examine the exact mechanisms by which metformin elicits its sexually dimorphic analgesic effects, especially with regard to its potential role as an epigenetic modulator. Should metformin be found to increase the levels of KDM6A/global H3K27me3 levels in females with a nerve injury, the outcome may not be favorable, given the link between KDM6A and autoimmunity.

### The Y chromosome

Unlike genes on the X chromosome, all Y-linked genes are expressed and, apart from duplicated genes, hemizygous, except in cases of aneuploidy. An evolutionary reconstruction across mammalian species suggests that preservation of specific portions of the Y chromosome over time did not occur randomly [266]. Rather, the gene content of the Y chromosome has become selectively specialized in order to maintain the ancestral dosage of homologous XY gene pairs that function as key regulators of transcription, translation, and protein stability in a range of tissues. Seventeen ancestral genes on the human Y chromosome have survived to the present day, with 4 (*HSFY*, *RBMY*, *SRY*, and *TSPY*) encoding isoforms that have functionally diverged from their X-encoded homologs (*HSFX*, *RBMX*, *SOX3*, and *TSPX*) to drive male reproductive development or gametogenesis [266]. In mammals, the *SRY* gene is the main driver of male development. However, even ubiquitously expressed human ancestral genes exhibit subtle functional differences from their X-linked homologs. In particular, 8 regulators of transcriptional activity that are present in numerous human tissues, including *DDX3X/Y*, *EIF1AX/Y*, *KDM5C/D*, *RPS4X/Y*, *TBL1X/Y*, *USP9X/Y*, *UTX/Y*, and *ZFX/Y*, exemplify a biochemical sexual dimorphism that directly originates from genetic differences between the X and Y chromosomes. Relevantly, several of these genes (*EIF1A*, *UTX*) have been implicated in chronic pain. Therefore, the Y chromosome may play as yet under-appreciated roles in broader sex differences that influence more processes than testis determination and spermatogenesis, impacting normal physiological functions as well as pathology. Research is currently underway to examine whether male-pattern neural development is a direct consequence of Y chromosome–related gene expression or an indirect result of Y chromosome–related androgenic hormone production [267].

It will be of future interest to investigate Y-linked genes in chronic pain conditions, which is currently understudied. One example that may shed light on the link between the Y chromosome and pain is Swyer syndrome, also referred to as 46,XY complete gonadal dysgenesis. Affected individuals have the typical male karyotype of one X and one Y chromosome per cell, but present with female reproductive structures, experiencing hormonal imbalances at puberty. While the Y chromosome cannot support the process of sexual differentiation and testes development, its partial function nevertheless lowers estrogen levels [268]. Depending on the genetic cause (primarily due to mutations in *SRY*, *DHH* (desert hedgehog), *MAP3K1*, or *NR5A1*), one of the co-morbidities of Swyer syndrome is neuropathy. Interestingly, the protein produced from *DHH* plays a role not only in male sexual development but also in the formation of the perineurium, the protective membrane around each bundle of fibers within a nerve [269]. Although rare, patients with 46,XY gonadal dysgenesis may present with chronic progressive motor and sensory polyneuropathy [270, 271].

### Sex chromosomes and immune cells

An immune response is fundamentally shaped by the X chromosome, which harbors a plethora of genes involved in this process (reviewed in [252, 272]). Reactivation of normally inactive regions of the X chromosome can lead to the breakdown of immune tolerance in females (reviewed in [272]). In a recent study, the transcriptome of 11 immune cell types (B lymphocytes (both B1A and B2 cell types), CD4^+^ and CD8^+^ T lymphocytes, dendritic cells, γδ T cells, granulocytes, macrophages, natural killer cells, natural killer T cells, and Treg cells) was profiled in 92 female and 91 male mice [171]. Expectedly, *Xist* and *Eif2s3y* were differentially expressed between the sexes in the majority of these cell types. With the exception of higher male expression of autosomal *Rps17* (40S ribosomal protein S17) in Treg cells, 41 other autosomal cell type-specific genes differentially expressed between the sexes were limited to macrophages [171]. Twenty six of these were more highly expressed in female macrophages, including genes involved in the complement system, particularly *Fcgr2b* (encoding FcγRIIB; inhibitory) and *Fcgr3a* (encoding FcγRIIIA; activating), which were discussed earlier in this review.

### Open chromatin in immune cells

During transcription, the chromatin in particular genomic regions becomes more accessible, allowing the required transcriptional machinery to assemble within a target gene regulatory region. An analysis of open chromatin regions may therefore be informative with regard to the regulatory status of particular cell types. Gal-Oz et al. analyzed open chromatin regions in murine male and female B cells, CD4^+^ T cells, and macrophages [171]. All female-specific open chromatin regions mapped to the X chromosome, including *Eif2s3x* (in macrophages), *Kdm6a* (in macrophages and CD4^+^ T cells), and *Xist* (in macrophages and CD4^+^ T cells), with all three loci already known to escape X inactivation [273]. In male macrophages, three autosomal open chromatin regions emerged, including the loci associated with *Bckdhb* (2-oxoisovalerate dehydrogenase subunit beta, mitochondrial), *Ift74* (intraflagellar transport protein 74 homolog), and *Ncam2* (neural cell adhesion molecule 2). The latter belongs to the immunoglobulin superfamily of cell adhesion molecules that contribute to homophilic trans-interactions [274]. In addition, differential open chromatin regions associated with unique genes were identified in macrophages and CD4^+^ T lymphocytes, the majority being female-specific.

The most prominent differentially accessible region was the X-linked locus harboring the functional intergenic repeating RNA element (*Firre*). This long non-coding RNA escapes, as well as helps maintain, X inactivation by anchoring the inactive X chromosome to the nucleolus [273]. It also establishes trans-chromosomal associations, recruiting specific loci on different chromosomes to its own transcription site [275]. In human CD4^+^ T cells, *Firre* is sex-specifically regulated due to greater enhancer activity in females than in males [276]. It was also recently shown that *Firre* may be regulated by NF-κB signaling and that it controls the expression of several macrophage-associated pro-inflammatory genes through post-transcriptional mechanisms [277].

Based on studies focused on identifying key sexual dimorphisms in the transcriptome of specific immune cell populations, evidence continues to emerge that supports an enhanced female potential to withstand immune challenge compared to males. This appears to stem from highly activated immune pathways that are primed even prior to pathogen exposure or in response to an injury, imparting heightened “immune alertness.” This priming would provide an advantage in combatting infectious diseases, but may come with the price of increased susceptibility to autoimmune conditions, which is evidence-based. But why would females have a hyper-alert immune system, if it may also contribute to pathologies such as persistent pain in response to certain types of nerve injury?

## The hyper-alert female immune system: recent hypotheses

### The pregnancy compensation hypothesis

One recently suggested hypothesis is that the female immune system has, for millions of years, been continuously prepared for the presence of a placenta, even in its absence. A recent opinion piece proposed that the requirement for females to compensate for unique immune system activity accompanying pregnancy was ancestrally guided [278]. Heritable variations in sex chromosome gene content and dosage have shaped the specialized immune function of pregnant females to ensure survival in the presence of an immunologically challenging placenta, with sex hormones directly impacting this process. This line of thinking may help to more broadly explain sex differences in immune function and the implications for associated pathologies, including pain.

The main premise of the pregnancy compensation hypothesis is that all placental mammals, including women, evolved to support high parity across a lifespan [278]. In keeping with this notion, women in hunter-gatherer populations commonly bore up to 12 children. Although there are risks associated with pregnancy, female physiology has evolved accordingly via key immune system adaptations. Natri et al. propose that this evolutionary process was necessary to counter the influence of the placenta, which signals the maternal immune system to alter its normal activity, thereby ensuring that the developing fetus is not rejected as “foreign” [278]. The placenta itself has the potential to be detected as a foreign organ, given that one of its two components, the chorion frondosum, develops from the blastocyst. The placenta and fetus are therefore treated as sites of immune privilege, with both engaging mechanisms that result in maternal tolerance. One such mechanism is placental secretion of phosphocholinated neurokinin B [279], which has been speculated to provide a “cloaking” system to the placenta, given that phosphocholines are also used by parasitic nematodes to evade host detection (reviewed in [280]). In addition, fetal small lymphocytic suppressor cells are able to inhibit maternal cytotoxic T lymphocytes by blocking their response to IL-2 [281]. However, dampening specific immune responses could have adverse effects, rendering women sensitive to pathogens with a potential negative impact on the developing fetus. As a compensatory mechanism, a woman’s immune system is primed throughout adulthood to constantly scan for pathogens, even during immune quiescence associated with pregnancy [278]. When continuous parity no longer occurs, which is currently the case in many Western countries, the placental “pushback,” which a woman’s immune system has evolved to anticipate, is absent. In this case, the female immune system is over-primed without purpose, and consequently, aberrant responses occur, which may be why the development of autoimmune diseases is on the rise in females.

### VGLL3

The pregnancy compensation hypothesis is not the first to explain why the incidence of autoimmunity is, in general, higher in women than in men. As an example, it has recently been found that women express higher epidermal levels of a putative transcriptional co-factor, vestigial like family member 3 (VGLL3), independent of biological age and gonadal hormone status [282]. VGLL3 exhibits female-specific nuclear localization, suggesting that it plays a key role in sexually dimorphic transcriptional regulation of its target genes. Its knockdown in vitro results in decreased expression of select female-biased immune transcripts, including B cell–activating factor (*BAFF*; also known as B lymphocyte stimulator and TNFSF13B), the target of belimumab, the only biologic currently used to treat systemic lupus erythematosus [283]. In contrast, men who suffer from this autoimmune condition demonstrate upregulated expression and nuclear localization of VGLL3 in their inflamed epidermis [282]. Furthermore, skin-directed over-expression of *Vgll3* in female rodents causes systemic autoimmunity that affects other organs, with symptoms resembling those observed in patients with systemic lupus erythematosus [284]. B cell expansion, autoantibody production, and immune complex deposition that ultimately contribute to tissue damage were all found to be engaged. Upregulated BAFF and chemokine (C-X-C motif) ligand 13 (CXCL13, also known as B lymphocyte chemoattractant) occurred as a consequence of over-expressed *Vgll3* in females, further implicating it as a driver of sex-specific autoimmunity [284]. The underlying driver of female skin cells expressing higher levels of VGLL3 is unknown, although Bili et al. speculate that, over the course of evolution, females have developed stronger immune systems at the cost of increased risk for autoimmune disease, which is along the lines of what Natri et al. propose with the pregnancy compensation hypothesis. It will be of considerable interest to examine VGLL3 and its immune target genes in the context of chronic pain. Interestingly, according to a research survey developed on behalf of the American Migraine Prevalence Prevention Advisory Group, among patients suffering from migraine-associated pain, cutaneous allodynia is more common in women, those with a higher body mass index, and those who are disabled or depressed [285], and tactile allodynia is a common symptom in patients coping with sciatica [286].

## Sex hormones

While chromosomes may shape male and female responses to pain, gonadal hormones have long been under investigation for their contribution to nociceptive signaling [287]. The general current consensus is that testosterone provides anti-nociceptive effects, clinically and in animal models of pain, with estrogen able to act both in an analgesic and hyperalgesic manner [288–290]. In mice, the sex chromosome complement, together with gonadal sex, influence the development of nociceptive signaling pathways as well as responses to analgesic drugs [214].

With regard to cross-talk between neurons and the immune system, the complex and seemingly paradoxical effects of estrogens have been extensively reviewed elsewhere [4, 291]. In the context of the variable effects elicited by 17β-estradiol and its metabolites on immune responses and repair systems, as well as the hypothalamic-pituitary-adrenal axis, the sensory nervous system, and the sympathetic nervous system, the consensus that has emerged is that the stimulus, cell type, target organ, microenvironment, reproductive status, concentration, receptor expression, and intracellular metabolism may all affect estrogen-mediated anti- and pro-inflammatory functions (reviewed in [292]). Estrogens play immunosupportive roles in trauma/sepsis, also accelerating the course of autoimmune diseases [293], and estradiol enhances specific T cell activity in female mice [294]. This is also the case in women, as post-menopausal estrogen deficiency has been linked with changes in T cell activation profiles [295].

Compared with estrogens, less is known regarding the effects of androgens on neuroimmune modulation. In male mice, the immunosuppressive actions of testosterone may be dampening the recruitment and activation of specific populations of T lymphocytes [294]. Clinical evidence supports that testosterone may protect against autoimmune disease [293, 296]. In men, androgen deficiency stemming from hypogonadotropic hypogonadism and Klinefelter’s syndrome (XXY) is associated with an increased risk of autoimmune disease. For example, in patients with Klinefelter’s, an 18-fold increase in the incidence of systemic lupus erythematosus has been reported, with clinical remission occurring in response to androgen therapy [297]. Testosterone deficiency also increases autoimmune disease-modeled activity in orchidectomized mice [298, 299], and androgen therapy improves male survival in a mouse model of systemic lupus erythematosus [300]. It is now generally accepted that testosterone is immunosuppressive, with this effect influencing sex differences in pain (reviewed in [156]).

### Sex hormones and opioid analgesia

Opioids including morphine are generally effective analgesics that are clinically applied to manage acute moderate to severe pain arising from injury, surgery, or cancer. However, tolerance associated with dose escalation and adverse effects (constipation, dependence, nausea, opioid-induced hyperalgesia, respiratory depression, and vomiting) are significant limiting factors in their use [301, 302]. As the “gold standard” opioid, morphine provides analgesia primarily via activation of spinal and supraspinal mu-opioid receptors (μ-OR), altering nociceptive signaling [303–307]. Morphine may also act peripherally on primary afferents [308, 309]. In preclinical studies, peripheral opioid receptors appear to potentiate anti-nociception, particularly in the context of inflammatory and neuropathic pain [303, 310–314], and it has been suggested that these peripherally expressed receptors may induce less adverse side effects than modulating their central counterparts [308]. However, under inflammatory conditions, spinal and supraspinal opioid receptors are the main modulators of anti-nociceptive responses [315–317], and the significance of peripheral opioid analgesia remains under debate.

Paradoxically, opioids such as morphine also exacerbate nociceptive hypersensitivity for weeks to months after treatment cessation in models of inflammatory and post-operative pain [318–323], as well as peripheral and centrally induced neuropathic pain [320, 321, 324–328]. It has been shown that morphine intensifies allodynia in a manner dependent on inflammatory signaling in the spinal cord, given that inhibiting either microglial activity or the action of pro-inflammatory cytokines during opioid administration prevents this effect [324–327]. Doyle et al. showed that within the periaqueductal gray of the rat, microglial activity provided a potential mechanism underlying the sexually dimorphic effects of morphine [329]. Interestingly, a recent study in male rats has shown that morphine, either administered pre-surgically or for 7 consecutive days commencing immediately after laparotomy, significantly prolonged post-surgical nociceptive responses [319].

A large body of literature covers sex hormone–regulated differences in opioid receptor–mediated anti-nociception. μ-OR is required for opioid-induced analgesia in males, while the kappa-opioid receptor (κ-OR) plays a predominant role in females [330–332]. κ-OR-mediated anti-nociception involves heterodimerization with μ-OR, followed by recruitment of the endogenous opioid peptide, dynorphin, with estrogen and progesterone regulating the anti-nociceptive conformation of these heterodimers [333]. Spinal expression of κ-OR varies across the estrous cycle, with its lowest receptor density correlating with low estrogen levels [334] and increased nociception [335]. Indeed, it has been clinically shown that women require higher concentrations of morphine than men to produce similar post-surgical analgesia [336, 337].

Opioid receptors are expressed on immune cells, including B and T lymphocytes, granulocytes, macrophages, and monocytes [338], and endogenously produced opioids modulate T cell proliferation and cytokine production [339]. In the context of nerve injury and inflammation, peripheral T lymphocytes have the capacity to release anti-nociceptive endogenous opioids, reducing inflammatory and neuropathic pain [309]. The specific population thought to be involved is CD4^+^ T lymphocytes [340].

Morphine can bind to TLR4 [341], which localizes primarily to microglia [151]. Its microglial interaction reduces the analgesic efficacy of morphine [342], particularly in females [329]. Morphine also binds to T cells [343], and Rosen et al. further investigated the modulation of exogenous opioid analgesia by these particular cells in males and females [344]. Based on a T cell–deficient acute inflammatory pain model, this study showed that sexual dimorphic opioid analgesia was lost in nude animals, but was restored upon injection of CD4^+^ T lymphocytes from immunocompetent donors [344]. In addition to displaying significantly higher baseline nociceptive sensitivity in the absence of nerve injury, various strains of T cell–deficient mice exhibited reduced morphine-mediated inhibition of inflammatory and thermal nociception. This work suggests that T cells may drive sex differences in anti-nociceptive opioid-mediated analgesia [344]. In particular, the T cell population relevant to this latter effect appears to be CD4^+^ lymphocytes, as their adoptive transfer into nude mice rescued baseline nociception as well as morphine analgesia, which did not occur upon transfer of CD8^+^ T cells.

### Sex hormones and microglia

A recent review summarizes the effect of sex hormones on microglia in the context of brain injuries, which provides relevant insights into the roles of estrogen and testosterone in the regulation of these cells in health and disease [345].

Microglia express estrogen receptor (ER)α and ERβ [346, 347], with estrogen significantly inhibiting their LPS-induced production of pro-inflammatory cytokines, also blocking the proliferation and activation of these cells in culture [347, 348]. The estrogenic modulation of microglial function has recently been reviewed [349]. In a model of Parkinson’s disease, in which microglial activity plays a prominent role, it has been shown that, while both the loss of murine dopaminergic neurons in the substantia nigra and LPS-induced microglial activation increased with age in both sexes, these effects were more pronounced in males [350]. In support of the notion that ovarian hormones such as estrogen play a role in these responses, bilateral ovariectomy abrogated the protective effect imparted against age- and LPS-induced microglial activation, with 17β-estradiol treatment of ovariectomized female mice reversing this effect [350]. In addition, pretreating cultured murine microglial cells with 17β-estradiol prior to LPS stimulation inhibited the expected increases in TLR4 and TNF-α levels. The effect of 17β-estradiol on the inward-rectifying K^+^ channel Kir2.1 was also examined in vitro, demonstrating a reduced probability of the channel being in an open state [350]. Kir2.1 is constitutively expressed in microglia and macrophages, serving as a means to maintain a negative membrane potential to regulate calcium influx and subsequent activation-associated microglial signaling [351–353]. Wu et al. concluded that age- and inflammation-associated activation of microglia is attenuated by ovarian estrogen, via its inhibitory action on Kir2.1 [350].

With regard to neuropathic pain, a recent study electrophysiologically examined the potential role of microglial K^+^ channels in a spared nerve injury model [354]. A notable increase in the expression of the Kir2.1 ion channel as well as Kir2.1-mediated inward currents associated with hyperpolarization of the resting membrane potential occurred in microglia two days post-injury, which was not observed in naive animals or at later post-injury time points. The electrophysiological changes coincided with the peak of microglial proliferation that takes place following a peripheral nerve injury, suggesting that microglial Kir2.1 may be an important therapeutic target with sexually dimorphic treatment outcomes [354].

Microglia also respond to androgens. Dampening mechanical allodynia using minocycline or a pharmacological blocker of P2X4 to inhibit the function of spinal microglia is limited to male mice, in which nerve injury has been reported to upregulate spinal P2X4 expression [155]. This sex-specific response was shown to depend on testosterone, as minocycline failed to inhibit allodynia in gonadectomized males while reducing allodynia in females treated with testosterone [155].

### Neurosteroids

Importantly, central and peripheral nerves themselves have the cellular machinery to synthesize and metabolize steroid hormones that are classically associated with the gonads (reviewed in [355]). In addition to nerves having the enzymatic capacity to support their production (reviewed in [356]), the intracellular androgen receptor (AR) is expressed in the rat sciatic nerve [357, 358] and steroid receptors for estrogen and progesterone have been detected in the rat sciatic nerve and Schwann cells [359–361]. Through their activation, androgens, estrogens, and progesterone, as well as their derivatives, may influence the development and function of the PNS and CNS, with these same molecules also modulating the activity of neurotransmitter receptors (NMDAR, GABA receptors) and non-classical neurosteroid receptors (for example, sigma 1 receptor) (reviewed in [356, 362]). In experimental models of peripheral neuropathy, neurosteroids, primarily testosterone and progesterone, largely act in a protective manner [363–366], playing a functional role in processes such as peripheral Schwann cell proliferation and myelination (numerous studies detailing the latter are reviewed in [356]). The concept that neuroactive steroids synthesized in the PNS exert neuroprotective actions has been reviewed in the context of their therapeutic application in treating peripheral neuropathy stemming from diverse causes, including aging, chemotherapy, diabetes, and physical nerve injury [367]*.* Interestingly, intraperitoneally administered progesterone fully reversed the nociceptive behaviors of male rats in which the peripheral sciatic nerve was impinged, with only partial recovery obtained in females [39]. It would be of interest to examine whether this sexually dimorphic response could be related to progesterone-mediated repair of myelin via Schwann cells, particularly given that progesterone is a precursor of testosterone. In a study by Caruso et al., neuroactive steroid levels were measured in the sciatic nerve, various CNS regions (the cerebellum, cerebral cortex, and spinal cord), and in the circulation of male and female intact and short- or long-term gonadectomized rats, resulting in distinc outcomes [368]. Post-gonadectomy, changes in neuroactive steroid levels in the nervous system did not necessarily reflect changes in plasma levels. Long-term gonadectomy led to altered PNS and CNS neuroactive steroids levels which, in certain cases, were distinct from those associated with short-term gonadectomy. Furthermore, the effect of gonadectomy on neuroactive steroid levels differed between the PNS and the CNS, as well as within the various CNS regions. Importantly, the effects of gonadectomy on neuroactive steroid levels in the nervous system were sexually dimorphic [368].

### Sex hormones and other specific examples: B lymphocytes and BAFF

As mentioned earlier in this review, B lymphocytes have been shown to infiltrate the impinged site of the sciatic nerve in an animal model of persistent neuropathic pain (Kim and Moalem-Taylor, 2011a), and B cells may be more prevalent in the DRG of male mice with a partial sciatic nerve ligation injury than in female counterparts [79]. B cells have also been implicated in other painful conditions. For example, in a study of classic interstitial cystitis, higher levels of B cell infiltration and clonal B cell expansion, which occurs in response to specific antigen exposure, were found to occur in patient-derived tissues [369]. B cells are also involved in TLR signaling, with TLR-mediated signals playing a role in both the removal and the activation of autoreactive B cells [370]. In addition, B cells have been implicated in nociceptive sensitization in a murine model of complex regional pain syndrome [371], and their role in chronic inflammatory conditions such as osteoarthritis is well known [372]. A recent review has suggested that B cells contribute to the autoimmune development and progression of multiple sclerosis by regulating T cell production and the antigen-presenting complex [373]. However, compared with other immune cell types, B cells remain a relatively understudied population in the context of persistent peripherally induced pain.

#### Testosterone and BAFF

In men with hypogonadotropic hypogonadism and Klinfelter’s syndrome, higher than normal circulating B cell counts are lowered in response to exogenous testosterone administration [374, 375]. Relevantly, in the bone marrow, testosterone suppresses B lymphopoiesis [376], and knockout of the murine AR in males increases the number of hematopoietic B cell precursors [377]. Furthermore, in men, testosterone and the AR suppress splenic B cell numbers [376] via an independent mechanism, potentially through the downregulation of BAFF [378]. This notion is supported by murine splenic BAFF deficiency resulting in a lack of mature B cells [379]. In addition, BAFF may play a role in autoimmune activity, with high levels promoting the survival of autoreactive B cells along with autoantibody production [380]. In healthy individuals, serum BAFF levels are higher in men presenting with lower-than-normal levels of testosterone, and a recent study has shown that testosterone directly regulates BAFF, as male mice lacking the AR have increased splenic *Baff* levels and B cell numbers, as well as higher circulating levels of this factor [381].

It will therefore be of considerable interest to examine the role of androgens and BAFF in the context of a peripheral nerve injury and nociceptive signaling. Interestingly, BAFF and its receptor (BAFFR) are also expressed in murine neurons, and a deficiency in BAFFR negatively affects neuronal survival [382]. Furthermore, impaired BAFFR-mediated signaling resulted in accelerated disease progression in an animal model of amyotrophic lateral sclerosis, a painful inherited condition characterized by gradual motor neuron degeneration. While the sex of the animals was not specified, neither knockdown of BAFFR in bone marrow nor genetic depletion of B cells affected this outcome, suggesting that BAFF-mediated signaling was occurring at the neuronal level [382].

#### Estradiol and BAFF

Estradiol suppresses bone marrow B lymphopoiesis [377, 383], and serum BAFF levels are higher in female compared with male mice [381]. Unlike the suppressive effect of testosterone, estradiol has been shown to upregulate BAFF at the mRNA level, as well as increasing the number of mature splenic B cells [383]. Similar to testosterone, estradiol acts on autonomic neuroeffector mechanisms, but it has been suggested that its effect reduces peripheral sympathetic nerve activity [384]. Therefore, the differential regulation of BAFF elicited by estradiol and testosterone, potentially driven by neural mechanisms that could involve neurosteroids, may contribute to sex difference in disease states in which this cytokine plays a pathogenic role.

### Sex hormones and other specific examples: TRPV1

TRP channels detect physical and chemical stimuli and promote painful sensations via nociceptor activation [385]. TRPV1 is activated by a range of stimuli (reviewed in [386]). It may act as a nociceptive mechanoreceptor [387] and has also been shown to be expressed along the entire length of C fiber neurons [388]. In mice, injecting the TRPV1 antagonist SB366791 at the site of a chronic sciatic nerve constriction injury or into the injured innervated hind paw dose-dependently alleviated mechanical and thermal sensitivity [389]. Intraperitoneal administration of this agent also potentiated the analgesic effects of systemic morphine in a murine model of bone cancer pain [390].

TRPV1 also plays a functional role in non-neuronal cell types, and a recent review has detailed its role in inflammation, immunity, and cancer [391]. It is expressed on CD4^+^ T lymphocytes [174] and microglia [392], controlling the cortical activation of the latter cell type [393]. TRPV1 has been associated with T cell antigen receptor–induced calcium influx and signaling as well as non-canonical (MHC-independent) T cell activation in a mouse colitis model, in which it promotes T cell responses to increase intestinal inflammation [174]. Treatment of activated CD4^+^ T cells with DPV576 (an aqueous mixture of nanodiamond and nanoplatinum) resulted in specifically decreased expression of TRPV1 [394]. Ghoneum et al. showed that this downregulation was accompanied by decreased IFN-γ secretion in capsaicin-activated CD4^+^ T cells [394]. T cells interact with macrophages and neutrophils, with these innate immune cells releasing cytokines that in turn modulate TRPV1 and TRPA1 [395, 396]. The expression profile of this ion channel therefore provides a possible link between nociceptors, microglia, and non-canonical TRP channel-mediated T cell activation.

#### Testosterone and TRPV1

It was recently shown that testosterone has a negative effect on *Trpv1* expression in a rat model of inflammatory pain associated with orofacial myositis [175]. Both its mRNA and protein levels were significantly upregulated in the trigeminal ganglia of castrated males three days after inducing inflammatory pain, as modeled by injecting complete Freund's adjuvant into the masseter muscle, with no effect on *Trpv1* expression in orchidectomized counterparts receiving testosterone replacement [175].

Whereas 17β-estradiol increases currents evoked by capsaicin in DRG neurons, promoting capsaicin-induced nociception [397], these effects are decreased by testosterone [398]. Testosterone has been shown to modulate the expression of neurotransmitter membrane receptors such as the cannabinoid receptor type 1 (CB1), with CB1-positive [399] and TRPV1-positive [175] rat trigeminal sensory neurons also co-expressing the AR [400]. Testosterone appears to play a key role in inhibiting TRPV1 expression in a rat chronic inflammatory pain model, providing a potential mechanistic basis for cytokine-hormone-neuron interactions [175].

#### Estradiol and TRPV1

Clinically, it has been reported that women respond to capsaicin-evoked pain more intensely than men [401]. Estrogen directly increases nociceptor excitability, reduces action potential thresholds, and facilitates TRPV1 activation in primary sensory neurons [402]. Furthermore, in ERα and ERβ knockout mice, the number of TRPV1 receptors is significantly lower than in wild-type counterparts [403]. Indeed, in female rats, estrogens upregulate *Trpv1* expression and exacerbate nociceptive responses in temporomandibular joints [177], trigeminal primary neurons [178], and the endometrium [176].

While there is evidence for increased TRPV1 channel activation in response to estrogen administration, the mechanism of this interaction remains unclear. Mechanical hyperalgesia induced by TRPV1 activation is believed to be mediated by G protein–coupled estrogen receptor 1 (GPER1), which is regulated by the overall level of estrogen. Estrogen-mediated GPER1 activation results in a rapid increase in intracellular cAMP and calcium levels in sensory neurons [291], with elevated intracellular calcium stimulating protein kinase Cε to evoke pain sensitization by phosphorylating TRPV1 within its C-terminus [404].

## Conclusions

The biological origins of sex differences that affect the outcome of pain are complex. Truly “moving the dial” in chronic pain research, which, from a patient perspective, will require fundamentally improving therapeutic interventions to represent precision medicine for men and women, will require additional focused research. One approach is through direct manipulation of gonadal hormones in relevant animal models of both sexes, at different life stages. Such an undertaking will determine how hormonal influences interact to modulate not only inflammation, but sustained neuroimmune cross-talk, providing a better fundamental understanding of hormonal roles in the development, maturation, and dysregulation of nociceptive circuits.

Gonadal steroids elicit distinct effects on pain responses and analgesic efficacy in adults, and perinatal dimorphisms in testosterone levels produce enduring organizational differences in males and females. Research supports that immune-triggered conditions exhibit a sex bias in children prior to the onset of puberty [405, 406], reinforcing that an exploration of sexually dimorphic facets that reach beyond gonadal hormones to include genetic and epigenetic processes will be required to fully understand pain chronification and to optimally treat persistent pain in men and women. Genes mapping to the X and Y chromosomes are emerging as important players, with the neuroendocrine system modulating neuroimmune crosstalk stemming from the sex chromosome complement. Sexually dimorphic mechanisms associated with increased expression of particular transcripts in patients who develop persistent pain in response to nervous system trauma are currently not well understood. Further research focused on the role of transcripts that escape X chromosome inactivation is needed to examine the consequence of low *XIST* levels, which could allow certain X chromosome-associated genes to escape inactivation in conditions involving persistent pain, including unresolved sciatica.

## Perspective and significance

There are clear similarities between chronic pain and autoimmune conditions. Given that the incidence of both pathological states is generally higher in women, it is worth considering that persistent nociceptive hypersensitivity itself may be the consequence of significant sexually dimorphic perturbations that are rooted in immune tolerance. Novel insights will only be gained by considering how these homeostatic perturbations as a whole lead to pain chronification, which may involve very different evolutionarily conserved biological processes in males and females. Examining chronic pain as an “autoimmune disease,” particularly in women, may broaden the scope for novel chronic pain therapeutics.

## Data Availability

Data sharing not applicable to this article as no datasets were generated or analyzed during the current study.

## Abbreviations

AR: Androgen receptor
BAFF: B cell–activating factor
BAFFR: B cell–activating factor receptor
CGRP: Calcitonin gene-related peptide
CB1: Cannabinoid receptor type 1
CNS: Central nervous system
CCL2: Chemokine (C-C motif) ligand 2
CNTF: Ciliary neurotrophic factor
JNK: c-Jun N-terminal kinase
DRG: Dorsal root ganglion
ERK: Extracellular signal–regulated kinase
ER: Estrogen receptor
EIF2: Eukaryotic initiation factor 2
FcγR: Fc gamma receptor
Firre: Functional intergenic repeating RNA element
GPER1: G protein–coupled estrogen receptor 1
IgG: Immunoglobin G
IFN-γ: Interferon gamma
IL: Interleukin
κ-OR: Kappa opioid receptor
LIF: Leukemia inhibitory factor
LPS: Lipopolysaccharide
KDM6A/UTX: Lysine-specific demethylase 6A
MHC-II: Major histocompatibility complex II
MMP-9: Matrix metallopeptidase 9
MAPK: Mitogen-activated protein kinase
μ-OR: Mu opioid receptor
NGF: Nerve growth factor
NMDAR: N-Methyl-d-aspartate receptor
NF-κB: Nuclear factor-κB
PNS: Peripheral nervous system
RNAseq: RNA sequencing
STAT3: Signal transducer and activator of transcription 3
SH2D1A: SH2 domain–containing protein 1A
Th: T helper
TLR: Toll-like receptor
TGF: Transforming growth factor
TRPV1: Transient receptor potential vanilloid 1
Treg: T regulatory
TNF-α: Tumor necrosis factor alpha
VGLL3: Vestigial-like family member 3
XIST: X-inactive specific transcript
YY1: Ying Yang 1
